# An assessment of a performance-based management agreement initiative in Ghana’s health service

**DOI:** 10.1186/s12913-018-3810-6

**Published:** 2018-12-27

**Authors:** Edmund Wedam Kanmiki, Ben Owusu Smith Bempah, John Koku Awoonor-Williams, Ayaga A. Bawah, Selassi Amah d’Almeida, Kassem M. Kassak

**Affiliations:** 1 Regional Institute for Population Studies, University of Ghana, Accra, Ghana; 2 Policy, Planning, Monitoring and Evaluation Division, Ghana Health Service, Accra, Ghana; 3 World Health Organization, Ghana Country Office, Accra, Ghana; 4 Department of Health Management and Policy, Faculty of Health Sciences, American University of Beirut, Beirut, Lebanon

**Keywords:** Performance agreements, Performance contracting, Performance management, Performance improvement

## Abstract

**Background:**

As part of its efforts to improve efficiency, accountability and overall performance, the Ghana Health Service (GHS) introduced annual Performance-based Management Agreements (PMAs) in the year 2013. However, no assessment of this initiative has since been made in order to inform policy and practice. This paper provides an assessment of this policy initiative from the perspective of managers at various levels of service implementation.

**Methods:**

Mixed methods were employed. Questionnaires were administered to managers through an online survey (using Google forms). Descriptive and inferential statistical methods were used to analyze and present quantitative results while qualitative data was analyzed via thematic analysis.

**Results:**

The content and objectives of the PMAs were observed to be comprehensive and directed at ensuring high performance of directorates. Targets of PMAs were found to be aligned with overall health sector objectives and priorities. The directors felt PMAs were useful for delegating task to subordinates. PMAs were also found to increase commitment and contributed to improving teamwork and prudent use of resources. However, PMAs were found to lack clear implementation strategies and were not backed by incentives and sanctions. Also, budgetary allocations did not reflect demands of PMAs. Furthermore, directors at lower levels were not adequately consulted in setting PMAs targets as such district specific challenges and priorities are not usually factored into the process. Insufficient training of staff and lack of requisite staff were key challenges confronting the implementation of PMAs in most directorates. Weak monitoring and evaluation was also observed to significantly affect the success of PMAs.

**Conclusion:**

There is the need to address the weaknesses and improve on the existing strengths identified by this assessment in order to enhance the effectiveness of PMAs utilization in the Ghana health service.

## Background

Performance-based Management Agreements (PMAs) has been given global prominence in public sector reforms that aim at achieving better performance and accountability [1–5]. The intrinsic thrust of their application is the development of common organizational objectives and establishing frameworks of operational, technical and allocative efficiency for managers. PMAs are “contracts” or “agreements” that bind two parties; the organization doing the spending or those responsible for budget allocation and those responsible for providing services [6, 7].

In healthcare delivery, PMAs usually specifies the range, quantity and quality of services to be delivered by the provider during a specific period. This is in contrast to traditional approaches where the allocation of public finances is based on existing facilities and staff regardless of the quality and quantity of services they provide [6].

PMAs therefore provides an avenue for linking financial allocations to pre-defined health service outputs and outcomes and provides stimuli for implementing policies through the provision of incentives for improving the utilization, distribution and cost-effectiveness of health services [6, 8–10]. Through the clarification of roles and responsibilities of the parties involved, PMAs stimulate accountability for use of resources to achieve key performance targets. The application of PMAs also enhances “performance culture” in managers and their staff by getting them to innovate to improve performance [4, 6]. PMAs can also be used as a vehicle to promote equity and support for marginalized populations through health policies that ensure redistribution of resources for providers serving areas of greater needs [6, 11].

Despite the growing interest in performance-based agreements for improving the performance of public healthcare delivery [12, 13], recent studies have documented mix outcomes on the effectiveness of PMAs in improving health systems performance of low and middle-income countries [9, 14]. Issues of poor designs of PMAs, hasty adoptions, financial constraints, lack of domestic ownership among others have been identified to impede the success of PMAs in some developing countries [9, 14]. In view of the mix evidence on the effectiveness of PMAs, there is a call for more appraisals of the implementation of PMAs in health systems of developing countries [13]. This paper provides an empirical assessment of a performance-based management agreement initiative implemented in the Ghana Health Service from the perspective of health managers.

### Structure and features of performance agreement in the Ghana health service

In Ghana, Performance-based Management Agreements were first introduced in 1997, they were at that time targeted at the performance of senior civil servants such as chief directors in Ministries and Regional Coordinating Councils [15]. Their implementation was however beset with challenges of oversight roles of institutional boards and absence of clearly defined implementation framework. In view of this, they were later redesigned to place a focus on cultivating effective performance management culture that aligns organizational objectives, targets and outcomes to national development priorities and goals [15].

In response to a national agenda to ensure efficiency and accountability in the delivery of public services in Ghana [15], the Ministry of Health (MOH) in 2013 instituted the signing of annual non-legally binding PMAs for all senior and middle-level managers [3]. In this framework, the Director-General on behalf of the Ghana Health Service signs a PMA with the Minister of Health who represents the government of Ghana. The heads of the various divisions at the headquarters and regional levels also sign PMAs with the Director-General of the Ghana Health Service (GHS) and district directors of health also sign with their regional directors [3].

While the roles and responsibilities of the parties vary each year, generally, the Ministry of Health’s roles under the PMA are to ensure the provision of adequate financial resources for the sustenance, improvement and remuneration of staff for the provision of health services by the GHS. Also, the MOH provides funding through regular budgets, the health fund and other governmental and nongovernmental sources to augment internally generated funds of the GHS for all their operations. The Ghana Health Service (GHS) on the other hand has the responsibility for planning, organizing and administering comprehensive health services and ensuring access to these services at all levels. The GHS is enjoined under the PMA to develop mechanisms for equitable distribution of services and promote health, prevent and control diseases and promote the efficiency and development of health workers. For each year, specific targets are set for the GHS to attain, these targets are defined in the Health Sector Medium Term Development Plan (HSMTDP) and accompanying annual program of work and associated PMAs. The GHS performance is measured by its attainment of targets defined by the key sector-wide indicators such as increase in skilled delivery, reduction in child mortality and reduction in maternal mortality among others which are often stated in the performance agreements.

It should be noted that within the GHS, managers at the divisional, regional and district health directorates levels signs performance agreement of which the targets are aligned to the overall sector objectives. Despite the perceived interest and benefits associated with the PMAs, there have not been any empirical studies of this initiative within the GHS in order to document lessons to inform theory and policy recommendation. This study, therefore, assesses this initiative with the goal of providing plausible recommendations for policy and practice improvement.

### Structure of the Ghana health service

The Ghana Health Service (GHS) was formally created and delineated from the Ministry of Health in the year 1996 through the Ghana Health Service and Teaching Hospital Act [16]. Ghana Health Service is responsible for the management and organization of health services, with the exception of the Teaching hospitals and Military hospitals, all other public health facilities in Ghana are under the management of the GHS [17].

**Fig. 1:**
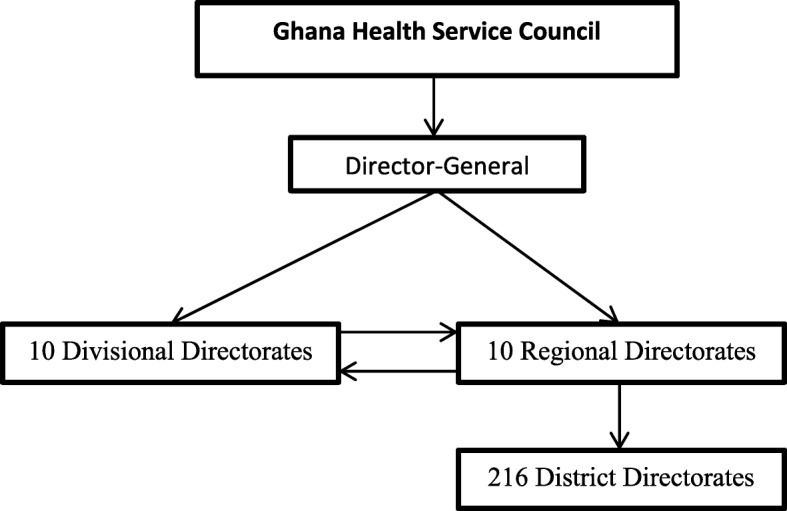
Structure of the Ghana Health Service

The GHS council is the highest authority in the Service and is responsible for providing both strategic and administrative advice. Next to the council is the Director-General who is the administrative head of the service. There exist ten divisional directorates at the national level whose power and authority is almost at the same level as the regional directors. Figure 1 shows the structure of the Ghana Health Service.

Regional directors are responsible for the day-to-day management and administration of health services at the regional level and are supported by a regional health management team [16]. At the district level, the district director of health services heads the district health management team and has overall responsibility over all health facilities and programs at that level [16]. There exists a management accountability relationship between the MOH, the GHS headquarters and the regional and district health administrations. Regions and districts are assisted in setting strategic plans with objectives and deliverables that are clear and quantifiable. Since 2013, Managers at all levels are required to sign PMAs with their supervisors to deliver on some specific objectives each year [3, 18]. This paper provides an assessment of this institutionalized annual PMAs within the GHS from the perspective of health managers. Results from this study are expected to contribute towards improving the process of PMAs in the Ghana Health Service.

## Methods

A combination of quantitative and qualitative approaches was used in this study as it is deemed to yield a better and in-depth understanding of the research problem than either of them alone [19].

### Data collection instrument

The research team developed a questionnaire based on a review of the literature [2, 4, 20–25], in which the following themes were identified as principal features of an effective system for implementing PMAs. (i) Context, rules and policies governing the PMAs (ii) Clarity of content and objectives of PMAs, (iii) Process involved in setting targets of PMAs, (iv) Capacity of directorates to implements PMAs, (v) Alignment of resource allocation with terms of PMAs and (vi) Perceptions of managers on usefulness of PMAs. Table 1 provides a description of these thematic areas as they influence the success of PMAs implementation.

**Table 1:** Description of Themes Influence on Success of PMAs

Thematic Area	Influences on the success of PMA implementation
Context, rules and policies governing the PMAs	The contextual factors that surround the signing and implementation of PMAs are critical to their successful implementation. The organizational culture, government politics, leadership style and capacities provide the context under which PMAs can be implemented to achieve intended objectives. It is important that clear rules, procedures and guidelines are provided to enhance the articulation and implementation of policy initiatives. These will facilitate effective negotiation and engagement with all stakeholders in the agreement [20].
Clarity of content and objectives of PMAs	One of the key objectives of PMAs is to clarify the roles and responsibilities of parties involved [27]. Health managers are enjoined to be abreast with the content and objectives of PMAs in order to facilitate successful implementation [23]
The processes involved in PMAs	The process for developing and implementing the performance agreement framework should be participatory involving all key stakeholders in the setting of goals, objectives, indicators and targets [21]. The process should further be interactive and parties involved must understand the importance of each stage of the process in order to contribute their quota to its success
Alignment of resource allocation with terms of PMAs	The provision of requisite resources that are aligned to PMA targets is for the success of PMA implementation [25]. In the absence of adequate resources, managers are limited in the extent to which they can implement activities that lead to the attainment of outputs and outcomes specified in the agreements [27]
The capacity of directorates to execute PMAs	The strengthening of institutional and staff capacities to implement performance agreements is one of the key ingredients for successful implementation [20]. It is also noted that due to the high transaction cost, government agencies must build the needed capacities for effective engagement, negotiation and implementation of performance agreements.
Managerial perception of its usefulness	Managerial acceptance and orientation to PMAs in terms of the right mindset and psychological disposition affect successful implementation [32]. Similarly, the perceptions of management about the useful impact of performance agreement instill a sense of commitment to its implementation. Managers who are committed to performance agreements use them to attract resources for implementation and can prioritize their activities to achieve the established objectives.

The questionnaire had two parts; the first part was fully structured (close-ended questions), it asked respondents to rank their level of agreement on a Four-point Likert scale (strongly agree, agree, disagree and strongly disagree) these covered various components of the themes. The second part of the questionnaire (qualitative) had two open-ended questions in which respondents were to write factors they deem to constraint the implementation of PMAs and factors that promote effective implementation of PMAs in the GHS. The survey instrument was developed by the authors and reviewed separately by two health system experts and appropriately revised before being used for the study. Also, a pretesting of the questionnaire was done at the headquarters level of the GHS which facilitated further refining of the instrument for the study.

### Data collection

Data collection took place over a 1-month duration (from 21st March 2017 to 20th April 2017).

Questionnaires were administered to directors of the GHS (divisional directors at the national headquarters, regional directors and districts directors) who are often signatories of PMAs were all targeted (a total 236 directors in all). The questionnaires were administered through Google forms; the link to the questionnaire was sent through emails to all divisional and regional directors. Regional directors were then asked to forward the emails with the link to district directors within their catchment areas (this is the official channel of communication within the GHS).

For the first part, respondents were to select their level of agreement on the various components of the thematic areas on a Four-Point Likert-scale. The second part (qualitative) involved open-ended questions in which participants were to write in their opinion on the PMAs implementation, its constraints and success drivers. In all 39 directors responded to the survey. Based on the fact that this study is qualitatively oriented with the presentation of descriptive statistics, the 39 respondents is deemed adequate to make informed analysis.

### Data analysis

The responses on components of the themes generated from various aspects of the PMAs initiative was rated. During the analysis, the score “Strongly disagree” and “Disagree” (1 and 2) represented aspects perceived negatively by respondents while “Agree” and “Strongly agree” (3 and 4) represented aspects perceived positively by respondents.

The SPSS software version 20 was used to aid in the analysis. Descriptive and inferential statistical methods were used to analyze and present quantitative results in tables and graphs. Qualitative data were analyzed using thematic analysis. The responses of the directors, which was collected through two open questions were organized into the different themes and presented alongside quantitative outputs to provide more in-depth understanding to the issues.

## Results

### Background characteristic of respondents

In all, 39 directors participated in the study, majority of them (74.4%) were district directors, and 15.4% were divisional directors based at the headquarters while regional directors and others formed 5.5% each. Only 10.3% of them had been in their positions for less than a year, 41% have held their positions between 1 and 3 years, 20.5% have been holding theirs for a period between 4 and 6 years and 28.2% have held their position for 7 years and above. In all 68.4% had previously held management positions prior to their current portfolios. 78.9% of respondents reported to having signed PMAs while 21.2% had never signed PMAs.

### Context: Rules and policies pertaining to performance-based management agreements

About 82.9% of directors either agree or strongly agree that they understand the PMAs protocols, 91.7% also alluded that PMAs are mandatory within the GHS. About 71.4% agree that the guidelines are comprehensive while 97.7% agree that it is binding to implement activities and strategies defined in PMAs. The average positive responses in this component is 84.4% culminating in a mean score of 3.1 out of 4 on the Likert scale (see Table 2).

**Table 2:** Summarized Results on Thematic Arears

Thematic Areas	Agree/Strongly Agree		Disagree/Strongly Disagree		Likert Mean Score out of 4
	Frequency	%	Frequency	%	
Understanding of the Rules, Policies & Norms pertaining to PMA within Ghana Health Service	30	84.4	6	15.6	3.1
Clarity of Content and Objectives	21	63.8	12	36.2	2.8
Processes Involved in Performance-based Contracting	18	52.9	16	47.1	2.5
Capacity of Directorates to Implement Agreements	20	59.5	13.5	40.5	2.6
Managerial Perception of Usefulness of PMA	18	56.3	14	43.7	2.5
Effect of PMA on Performance	22	66.7	11	33.3	2.8

### Clarity of content and objectives of PMAs

High percentage of respondents (90.9%) agree that the current PMAs have clear objectives, which are well explained and negotiated (79.4%), comprehensive (97.1), directed at measuring relevant performance of their directorates (100%) and are aligned to the overall sector objectives (97.1%).

However, participants were almost divided as to whether the PMAs outlines clear implementation strategies (58.8% agree while 41.2% disagree). A district director indicated that:

*“Performance agreements are so broad and do not give managers adequate information on the essentials. Not objective assessment*”

Majority of respondents (82.4%) agreed that their directorates have a work plan to guide the implementation of PMAs. On the issue of incentives and sanctions, as high as 84.8% were of the view that the current PMAs do not specify clears incentives and sanctions. To provide in-depth understanding to the issues, a district director who has been involved in signing PMAs for over 3 years lamented that;

“*Directors are not held accountable in instances of not achieving targets set in the agreements”.*

Also, a regional director who has signed the PMAs for about 5 years recommended that:

*“Performance agreements should be linked to incentives and sanctions. These should be determined through assessment of process indicators, consistency in performance improvement and not just the final output performance”*

From the results of both quantitative and qualitative analysis, it is evident that the current PMAs initiative does not include measures for the provision of incentives and sanctions where necessary.

### Resource availability for implementing performance agreements

**Fig. 2:**
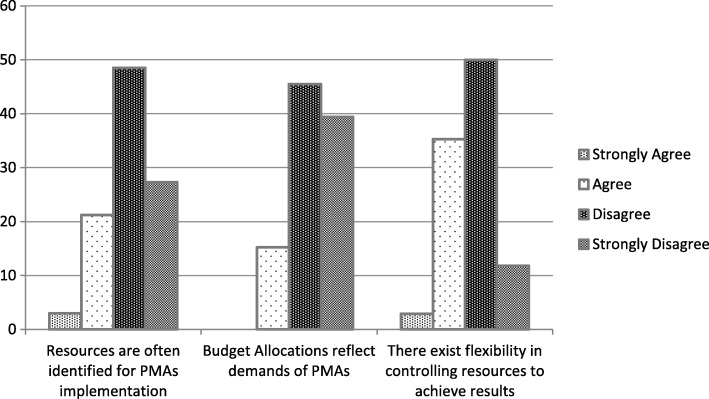
Resource Identification and Allocation for Implementing PMAs

As high as 75.8% of the respondents felt that resources are not usually identified for PMAs implementation and 84.8% feel budget allocations do not match the demands of the PMAs signed. Figure 2 illustrates this fact.

Results from the qualitative analysis provide more information on the resource allocation situation pertaining to performance agreements in the GHS. A divisional director who has participated in PMAs for three times pointed out that inadequate resource allocation is a major constraint to achieving targets of PMAs within GHS:

*“The problem with the performance agreements entered into has been the non-provision of resources clearly identified as required for implementation. It therefore makes it difficult to attribute outputs choked to the performance contract”*

Another divisional director who has also participated in PMAs for 3 years indicated that:

“*Agreements are signed without any identified financial commitment for execution”*

A district director also expressed worry about the non-provision of resource for achieving PMAs terms:

*“No resources whatsoever were given for executing the performance agreements; you must struggle to gather your own resources at times from personal and private sources just to achieve your objectives. Notwithstanding; if an objective is not achieved it is portrayed as if the Officer is not hard working or not up to the task.”*

Another divisional director said that;

*“The major challenge is resource allocation. Especially financial and human resources, we have to do a lot of monitoring and evaluation but we are limited due to this”*

From the foregoing, it is evident that the provision of resources for implementing PMAs is a critical challenge to establishing an effective system. From the qualitative accounts, a regional director highlighted the lack of flexibility in the use of resources to achieve the terms of the PMAs; this is also depicted in Fig. 2.

### Process involved in PMAs

About 75.8% of respondents are of the view that there is a well-structured process for PMAs while 24.2% disagree with that. A divisional director observed the inappropriate timing of the signing of the PMAs. The director stated that PMAs signings are sometimes done in the third quarter of the year thereby making the whole process not worthwhile.

As high as 76.5% of directors said they were not involved in the process of designing the PMAs framework. A district director mentioned the poor involvement of the district managers in the planning process. There was also the expression of worry with the imposition of targets from the national level, one district director concluded that:

*“District Teams should be allowed to identify their own targets rather than National selecting and compelling them to adopt or go by such priorities”*

A district director who participated three times in the PMAs said:

*“……district specific challenges are not considered and as such, the harsh, deprived districts are expected to perform just as those with better opportunities. Again, they are just sent to the directors without any prior discussions.”*

In terms of information flow, 35.3% of respondents felt adequate information is often not provided to them prior to the signing of agreements. These also attest to the poor or non-involvement of line managers in the process, which invariably affects the effectiveness of the PMAs.

Furthermore, 79.4% disagree that the process has an inherent system for rewarding outstanding performance. This is also a further attestation to the non-existence of incentives and sanctions for outstanding and poor performance respectively. Whiles 63.5% felt that PMAs are monitored and evaluated for policy and practice, 36.4% of respondents disagree to that. The qualitative account reveals that there might be weak monitoring and evaluation of PMAs within the system. A divisional director was of the view that:

*“The key challenge is lack of follow up and monitoring the agreement once signed”*

Another divisional director expressed a similar sentiment that:

*“No Feedback on performance evaluation or not much review is done on our performances”*

A district director advocated for a monitoring team at the regional level by expressing that:

*“……There is the need to have separate Monitoring team at the Regional level to monitor and supervise weak Districts who do not have adequate qualified personnel or due to maldistribution of staff…….”*

### Capacity of directorates to implement agreements

As high as 63.6% of directors stated that their outfits did not receive adequate training for implementing the PMAs. A district director who has participated in the PMAs process for 3 years observed that:

*“No detailed training was done for managers on performance agreements”*

Another district director who has also been involved five times has this to say

“….. *and inadequate training for staff to understand the elements of the annual performance appraisal system”*

Despite this, 73.5% of directors felt their directorates have adequate knowledge of the importance of PMAs. A district director in the qualitative account mentioned that PMAs are good managerial tools.

Directors were almost divided as to whether they have requisite staff numbers and mix to implement PMAs (51.5% agree that they have, while 48.5% disagree) this might suggest and indeed emphasis the disparities in staff distribution within the GHS. A divisional director was emphatic that inadequate staff with the right mix is a major bottleneck for effective implementation of PMAs. Many other directors alluded to this as a major constraint to implementation. Attention was also made to directorates’ lack of authority to hire and dismiss staff where necessary as a hider to achieving targets.

Another related matter to the capacity of directorates to implement PMAs was the competencies of health managers. In outlining the challenges of the current PMAs, A district director was passionate about the competence of people appointed to managerial position in the GHS. He has this to say:

*“employing people to managerial positions based on chain of degrees without necessarily seeking qualities of commitment and passion for output of such employees”*

He went further to write in block letters that;

*“THE SQUARE PEGS IN ROUND HOLES IS KILLING THE HEALTH SERVICE AND WITH TIME, NO TARGET WILL BE ACHIEVED IN THE PUBLIC HEALTH INTERVENTIONS UNLESS A SECOND LOOK IS TAKEN TO REVIVE THE DYING HEALTH SYSTEMS AT THE LOWER LEVEL”*

### Managerial perception of usefulness of PMAs

**Fig. 3:**
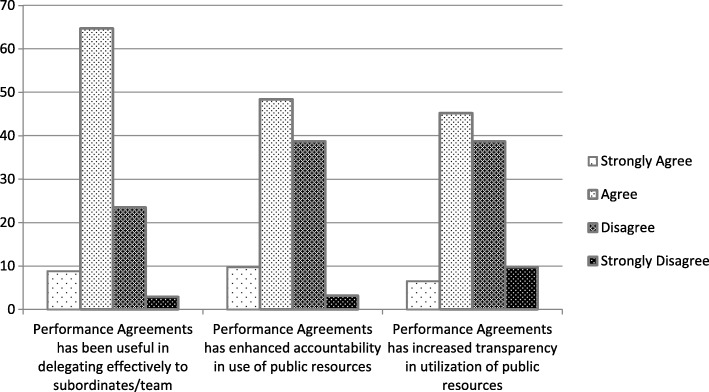
Managerial Perception of Usefulness of PMAs

Leadership perception of the usefulness of any intervention is very important in influencing their commitment to its eventual success. Figure 3 presents results of directors perception of the usefulness of PMAs, 73.5% of respondents agreed that PMAs have been useful in delegating effectively to subordinates. 41.9 and 48.4% of respondents disagree that the current PMAs has enhanced accountability and transparency in the use of public resources respectively.

To understand why directors are divided as to whether the current performance agreements have enhanced accountability, the qualitative accounts are examined. A district director with 5 years’ involvement in signing PMAs within the GHS said that:

*“Performance agreements should improve accountability and responsiveness of the system. However, Resources are not made available for management and staff to perform hence the difficulty in holding staff accountable to set objectives because we obviously failed to provide the needed working tools and funds”*

Another respondent suggested that in order to enhance accountability:

*“Performance agreements and appraisals should be integrated and mandatory at all levels to facilitate accountability”*

To elaborate further on the managerial perception of usefulness, a district director said that:

*“Performance agreements is a fantastic means of improving performance however, if resources are not provided, it becomes difficult to fairly assess performance. The Ghana Health Service needs to look at this and take steps to address it.”*

Other directors concurred that PMAs are good managerial tools. Indeed, no respondent advanced any negative issues on the usefulness of the PMAs in principle however, issues regarding the process of involvement and resources were highlighted as bottlenecks to effective implementation.

### Effect of PMAs on performance achievement

As high as 90.9% of respondents agree that PMAs has increased their commitment to achieving objectives and priorities of the service. 78.1% noted that it has contributed to improving teamwork. About 51.5% indicated that PMAs contributed to mobilizing resources for implementation and 67% of respondents disagree that PMAs improved the level of resource predictability.

Overall, while the current PMAs arrangement has positively impacted on commitment, teamwork and prudent use of resources, it has not been able to adequately improve resources mobilization and predictability, it’s contributing to improving monitoring and evaluation in the system is also not apparent.

## Discussion

This study seeks to assess the current Performance-based Management Agreement (PMA) initiative being implemented within the Ghana Health Service (GHS). The objective is to provide empirical evidence for improving the effectiveness of its implementation.

It is apparent from the results that directors of the GHS have a good appreciation of the rules and norms of the PMAs policy. According to Van Meter and Van Horn model of policy implementation, a key element for the success of any policy is for implementers to have a clear understanding of the policy standards and protocols [20].

Also, study respondents find the current PMAs to have clear and comprehensive objectives that are relevant and well aligned with overall health sector objectives. This is remarkable since clear and easily understood objectives facilitate the success of PMAs [25, 26]. Chhabra points out that managers should be provided with adequate education on the philosophy and purpose of the contracts/agreements [23]. In the same vein, Easterbrook-Smith brings out the fact that among the aims of PMAs is to clarify objectives to all key stakeholders [27]. However, both quantitative and qualitative results show that the current PMAs initiative does not include provisions for incentives and sanctions where necessary. Armstrong and Baron have outlined the need for providing rewards to top performers in PMAs. They suggest measures such as recognition, the award of medals and other materials based on the local context of organizations and departments [28].

None provision of resources for implementing PMAs was also observed. The provision of resources for implementing PMAs is critical to establishing an effective system. Studies have affirmed that good PMAs ensures the provision of adequate and timely resources for implementation [25, 26]. Indeed, in the absence of adequate resources, managers are limited in the extent to which they can implement activities including processes that lead to the attainment of outputs and outcomes specified in the agreements [27].

Participants also indicated that they were not often involved in setting targets for their directorates. Chhabra again asserts that the involvement of managers and their subordinates in the planning and designing of PMAs is important for successful implementation. He further criticized situations in which goals and targets are set by superiors and imposed on subordinates without their inputs [23]. According to Chhabra, people directly responsible for meeting the goals should be made to participate in goal-setting [23]. Armstrong is of the view that managers and subordinates must act as partners in developing the PMAs framework which should be continues and flexible [29]. It has been cautioned that there could be the possibility of unintended and undesirable outcomes such as goal displacement and strategic manipulation if targets are imposed on implementers [25].

Week monitoring and evaluation was also identified to impede the success of PMAs implementation. The importance of periodic evaluation of signed PMAs to ascertain the achievement of targets and objects spelled out in them cannot be overemphasized [26]. In detailing the key elements for the success of PMAs, Armstrong and Baron calls for periodic evaluation of staff or departments performance and achievements of the work plan stipulated in the PMAs [28]. This also provides an opportunity for identifying outstanding performers for recognition and possibly awards [25, 28].

The quality of some health managers was brought to question by some participants. Effective management and sound leadership are imperative for the success of any organization as well as the implementation of any initiative. Van Metter and Van Horn outlined how the disposition of leaders is paramount to sound implementation of reforms [20]. Sound implementation of PMAs requires skilled managerial abilities [24]. It is also noted that managerial skills for PMAs should be improved through formal training supplemented by mentoring and coaching [24]. It has been documented that institutions with specializes employees of the right mix and numbers achieve targets more than those with minimal training, hence the need to adequately enhance human resources situation of institutions [25].

It also emerged that directorates lack the authority to hire and dismiss staff where necessary which hider the achievement of PMA targets. Since health managers do not have sufficient control over both human and material resources in their jurisdictions and yet are made to sign PMAs to achieve targets with those resources, it raises issues of injustices. Previous studies have documented limited authority of managers to adversely affect the success of PMAs [30]. Efforts should therefore be made to enhance the authority of managers to control both human and material resources within their directorates.

Participants generally agreed that PMAs are useful managerial tools for improving performance especially for delegating responsibilities to subordinates although they were divided when it comes to the contribution of PMAs to improving accountability in GHS. Studies have shown that wherever PMAs were introduced, they increased the level of satisfaction, improved comfort and reduced tension and hostility between and among superiors and their subordinates [23]. Indeed application of PMAs has been found to improve service delivery in public institutions and led to public institutions being more customer and performance oriented [12, 22, 31–33]. PMAs has therefore been acclaimed as an important strategy for enhancing efficiency, effectiveness and customer satisfaction in organizations [25].

### Study limitations

Due to busy schedules, it was difficult accessing respondents, the participation of regional directors in particular was relatively low. Also, Performance-based Management Agreements/contracting is a broad area of study itself therefore not all aspects could be assessed in this study. These notwithstanding, the use of qualitative analysis to complement the quantitative assessments strengthens this study. Finding of this study are therefore reliable and are valuable for policy and practice improvement.

## Conclusion

This study has evaluated the current performance-based management agreements initiative of the Ghana health service from the perspective of health managers. It is evident that a combination of structural and contextual factors constrains the current PMAs initiative of the GHS from bringing about the required improvements to achieve performance targets.

Clear implementation strategies should be made available during the signing of PMAs, incentives such as awards, promotions, recognition among others, should be part of the process while sanctions should also be put in place to engender commitment to achievements of targets in PMAs. Importantly, budget allocations should reflect demands of PMAs and line directors should be involved in the process of setting targets for their directorates. Additionally, efforts should be made to provide adequate training for staff for achieving targets in PMAs. It is also important to strengthen monitoring and evaluation of the process to provide useful lessons and measures for correcting deviations during implementation.

This study contributes to the literature on improving public sector performance as it relates to many reform initiatives which are founded on the principles of new public-sector management. Specifically, it introduces the novelty to get the fundamentals right in addressing the context specific to each country implementing performance agreements in its public health sector.

## Abbreviations

GHS: Ghana Health Service
MOH: Ministry of Health
PA: Performance agreement
PMAs: Performance-based Management Agreements
RHMT: Regional Health Management Team
